# Editorial: Machine learning and applied neuroscience

**DOI:** 10.3389/fnbot.2023.1191045

**Published:** 2023-04-06

**Authors:** Wellington Pinheiro dos Santos, Vincenzo Conti, Orazio Gambino, Ganesh R. Naik

**Affiliations:** ^1^Department of Biomedical Engineering, Federal University of Pernambuco, Recife, Brazil; ^2^Faculty of Engineering and Architecture, Informatics Engineering, University of Enna Kore, Enna, Italy; ^3^Department of Engineering, University of Palermo, Palermo, Italy; ^4^Adelaide Institute for Sleep Health, Flinders University, Adelaide, SA, Australia

**Keywords:** Computational Neuroscience, neuroengineering, Computational Intelligence (CI), artificial neural network, applied neuroscience

A set of disruptive technologies, such as Computational Intelligence, Pervasive Computing or Internet of Things, Robotics and Biotechnology characterize the IV Industrial Revolution. These technologies allow for an increase in productivity and economic complexity, modernizing the production of high-added value goods and services in the leading economies of the globe but also opening up important opportunities for emerging nations in areas such as Neuroscience applications or Computational Neuroscience.

Evolutionary Computing and Deep Learning allow the construction of increasingly accurate expert systems with greater learning and generalization capabilities. When applied to Neuroscience, these advances open up more possibilities for understanding the functioning of the nervous system and the dynamics of nervous diseases, as well as constructing models of learning machines that are increasingly accurate and capable of dealing with complex problems, such as computer vision and complex pattern recognition tasks.

Advances in Computational Intelligence, especially new methods, algorithms, and computational architectures, allow the realization of complex tasks, such as the construction of customized diagnostic and treatment methods for diseases of the nervous system, the structure of intelligent prostheses, and more precise brain-machine interfaces; the identification of emotions and the diagnosis of mental disorders through automated analysis of signals of various types, and other applications.

This Research Topic is composed of the following contributions:

In “*Learning Transferable Push Manipulation Skills in Novel Contexts*,” Howard and Zito propose to learn a parametric internal model for push interactions to enable a robot to predict the outcome of physical interaction in novel contexts. They organized the learning process into two parts. First, the model learns from local contact models to represent the geometrical relations between the robot pusher, the object, and the environment. Afterward, the model learns a set of parametric local motion models to predict how these contacts change throughout a push. The authors validated their proposal using a simulated environment composed of a Pioneer 3-DX robot fitted with a bumper to predict a push outcome for an object in a novel context. According to the authors, both biased and unbiased predictors can reliably produce predictions in line with the consequences of a carefully tuned physics simulator.

In “*A Fusion Algorithm of Object Detection and Tracking for Unmanned Surface Vehicles*,” Zhou et al. propose a fusion algorithm of detection and tracking for unmanned surface vehicles (USV) targets. To solve the problem of vague features in the single-frame image, high-resolution and deep semantic information obtained through a cross-stage partial network. The anchor and convolution structure in the network has been improved given the characteristics of USV. The authors built a picture dataset and a video dataset to test the effect of detection, tracking, and fusion algorithm separately, proving the fusion algorithm's authority in this paper by increasing the success rate by more than 10%.

In “*Low-Light Image Enhancement Network Based on Recursive Network*,” Liu et al. propose a multiscale feature fusion image enhancement network based on a recursive structure. The network uses a dual attention module-Convolutional Block Attention Module. Using the inception model, the authors extended the U-Net model to form the multiscale inception U-Net Module, or MIU module for short. The learning of the whole network is divided into T recursive stages. The input of each stage is the original low-light image and the intermediate estimation result of the output of the previous recursion. The experimental results on several public datasets show that the proposed model can recover the details, increase the brightness of the imageimage's brightness better, and reduce image degradation compared with other state-of-the-art methods.

In “*Attention-Guided Multiscale Feature Fusion Network for Low-Light Image Enhancement*,” Cui et al. propose an Attention-Guided Multiscale feature fusion network (MSFFNet) for low-light image enhancement for enhancing the contrast and brightness of low-light images based on a single encoder and decoder for multiscale input and output images. Using a set of public datasets, the authors could demonstrate evidence that the proposed MSFFNet outperforms other low-light enhancement methods in terms of visual effects and metric scores.

Xiang et al., in “*Recent Machine Learning Progress in Lower Limb Running Biomechanics with Wearable Technology: A Systematic Review*,” present a systematic review of machine learning approaches in running biomechanics using wearable sensors. According to the authors, the deep learning model combining multiple CNN and recurrent neural networks (RNN) could extract different running features from wearable sensors and presents a growing trend in running biomechanics.

Ou et al., in “*An overview of brain-like computing: Architecture, applications, and future trends*,” present an overview of the progress and status of brain-like computing, focusing on the generally accepted and feasible brain-like computing models. Authors analyze the more mature brain-like computing chips, outline the attempts and challenges of brain-like computing applications, presenting some important comments on the future development of brain-like computing.

Finally, Jia and Liu, in “*Anomaly detection in images with shared autoencoders*,” propose a new machine learning model based on two decoders to share two encoders, respectively, forming two sets of network structures of encoder-decoder-encoder (EDE), used to map image distributions to predefined latent distributions and vice versa. The authors propose a two-stage training mode. The second stage employs the idea of generative confrontation to send one of the two groups of reconstructed vectors into another EDE structure to generate fake images and latent vectors. The proposed architecture, combined with unique training methods, could avoid the shortcomings of generative adversarial networks (GANs) and AEs, achieving competitive results regarding the state-of-the-art when validated on several publicly available image datasets.

We hope you enjoy our edited contributions.

## Author contributions

All authors listed have made a substantial, direct, and intellectual contribution to the work and approved it for publication.

